# The Latest Advances in Omics Technology for Assessing Tissue Damage: Implications for the Study of Sudden Cardiac Death

**DOI:** 10.3390/ijms26146818

**Published:** 2025-07-16

**Authors:** Raluca-Maria Căținaș, Sorin Hostiuc

**Affiliations:** 1 Department of Legal Medicine and Bioethics, Carol Davila University of Medicine and Pharmacy, 020021 Bucharest, Romania; rmcatinas@gmail.com; 2 National Institute of Legal Medicine, 042122 Bucharest, Romania

**Keywords:** sudden cardiac death, proteomics, metabolomics, transcriptomics, forensic science

## Abstract

Sudden cardiac death (SCD) is a major public health concern, being a leading cause of death worldwide. SCD is particularly alarming for individuals with apparently good health, as it often occurs without preceding warning signs. Unfortunately, traditional autopsy methods frequently fail to identify the precise cause of death in these cases, highlighting the need for advanced techniques to elucidate underlying mechanisms. Recent advances in molecular biology over the past few years, particularly in proteomics, transcriptomics, and metabolomics techniques, have led to an expanded understanding of gene expression, protein activity, and metabolic changes, offering valuable insights into fatal cardiac events. Combining multi-omics methods with bioinformatics and machine learning algorithms significantly enhances our ability to uncover the processes behind lethal cardiac dysfunctions by identifying new useful biomarkers (like cardiac myosin-binding protein C, acylcarnitines, or microRNAs) to reveal molecular pathways linked to SCD. This narrative review summarizes the role of multi-omics approaches in forensic diagnosis by exploring current applications in unexplained cases and the benefits of integrating merged techniques in otherwise negative autopsies. We also discuss the potential for developing personalized and preventive forensic medicine, the technical limitations of currently available methods, and the ethical considerations arising from these advancements.

## 1. Introduction

Sudden cardiac death (SCD) is one of the most important public health concerns, being a leading cause of death worldwide, with an estimated percentage between 7% and 18% of total deaths in the U.S. [1]. SCD is defined as an unexpected decrease within one hour of symptom onset or even without clinical prodrome, with myocardial infarction and coronary artery disease being the most frequently found underlying pathophysiological causes, especially in the elderly population [2,3]. SCDs affecting young adults (<40 years) are often identifiable, as their usual underlying causes determine structural changes, with entities like hypertrophic cardiomyopathy, dilated and restrictive cardiomyopathies, myocarditis, or coronary artery disease often being found [4,5]. These structural alterations are usually detected during autopsy, so the cause of death is well known. On the other hand, arrhythmogenic disorders (e.g., familial long-QT syndrome, short-QT syndrome, Brugada syndrome) have no structural counterpart to be observed in macroscopical and microscopical analyses, so that the autopsy and associated laboratory examinations would not be able to identify the cause of death postmortem, thus leading to approximately 40% of “negative autopsies” in SCDs among young individuals [5,6,7,8,9,10,11]. Given this huge number of cases where death remains unexplained, it is obvious that traditional autopsy and histopathology, though foundational, are insufficient due to important diagnostic gaps [12,13,14]. To overcome the limitations of conventional diagnostic methods, new molecular techniques and biomarkers have been increasingly studied to evaluate both antemortem and postmortem causes of SCD, where the rest of the tests are negative. With significant shifts amongst modern lifestyle and risk factors (hypertension, diabetes, obesity, smoking), as well as the challenges posed by the genetic predisposition in the occurrence of cardiovascular diseases, advances in biomolecular diagnostics have been made, particularly in proteomics, metabolomics, and transcriptomics, now offering promising avenues for identifying early, specific, and stable markers of myocardial damage [15,16].

This narrative review summarizes the latest research in molecular techniques, particularly in proteomics, metabolomics, and microRNA profiling, for evaluating tissue damage in the context of SCD and exploring its diagnostic and forensic implications. The included articles were chosen by searching the PubMed and Web Of Science databases for the following keywords in the title and abstract: “proteomics” OR “proteomic” OR “metabolomics” OR “metabolomic” OR “microRNA” OR “microRNAs” OR “miRNA” AND “sudden cardiac death” OR “sudden death” OR “cardiac death”.

**Figure 1:**
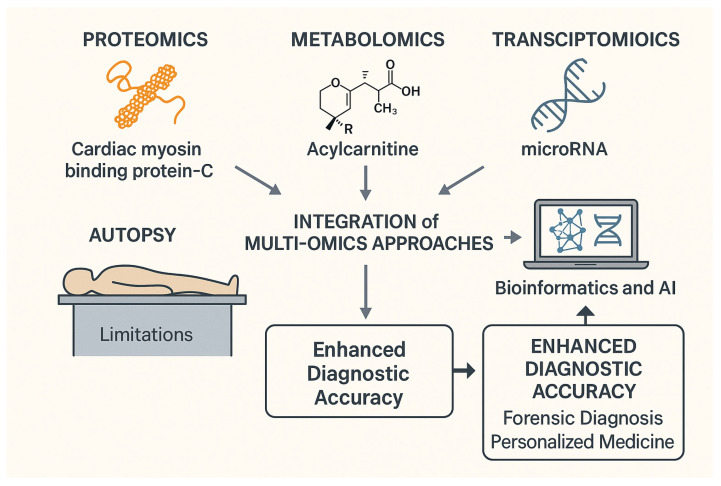
Multi-omics integration for forensic diagnosis and enhanced diagnostic accuracy.

Figure 1 summarizes our narrative review on integrating multi-omics technology to improve the accuracy of forensic diagnosis. 

## 2. Proteomics in Tissue Damage and Sudden Cardiac Death

Proteomics is the branch of omics involving proteome analysis—the complete set of proteins expressed by a cell, tissue, or organism in a specific physiological or pathological context. It plays a central role in characterizing protein expression, identifying diagnostic and/or prognostic biomarkers and therapeutic targets, as well as elucidating molecular mechanisms involved in various pathologies [17,18]. Multiple techniques undergo continuous technological advancements to obtain increasingly detailed information from proteomic samples. The most commonly used protein analysis methods are two-dimensional gel electrophoresis, liquid chromatography–mass spectrometry, sequential window acquisition of all theoretical mass spectra, protein microarrays, protein–protein interaction assays, and traditional imaging techniques, such as fluorescence and electron microscopy [19].

### 2.1. Proteomic Analysis Techniques

#### 2.1.1. Two-Dimensional Gel Electrophoresis (2D-GE)

Introduced since the 1970s and improved in the meantime, 2D-GE is one of the most robust and frequently used methods for comparative proteomic studies [20,21]. The technique separates proteins into two stages: in the first dimension by isoelectric point (pIs), and in the second by molecular mass. This will generate a two-dimensional profile of protein spots visible on gels that are stained afterwards with specific compounds (e.g., Coomassie Brilliant Blue, silver staining). The images obtained undergo a computer quantitative and qualitative analysis of protein spots between gels. The purpose of all of this is to compare the expression profiles between distinct experimental conditions. Although this technique has a lot of advantages, including low cost, high sensitivity, and compatibility with complementary techniques such as mass spectrometry, 2D-GE presents several limitations, such as experimental variability, gel deformations, spot overlap, and visual artifacts (streaks, noise, oversaturated or weak spots), which significantly affect the reproducibility of the results. Therefore, image preprocessing—such as geometric corrections, precise gel alignment, and optimization of spot detection through different methods—becomes essential to reduce systematic errors and artifacts as well as increase the accuracy of proteomic analysis. Integrated commercial software platforms are available for 2D-GE image analysis (e.g., PDQuest, ImageMaster2D, ProteomeWeaver), but these can be expensive and are often made for a particular market. Alternatively, a free software such as ImageJ can be used, but it has automation and large-scale analysis limitations [17,22,23,24,25,26,27].

#### 2.1.2. Liquid Chromatography Tandem Mass Spectrometry (LC-MS/MS)

LC-MS/MS is regarded as the gold standard method for large-scale proteomic analysis. The samples analyzed using this high-throughput technique undergo a complex process of denaturation, reduction, alkylation, and trypsin digestion, resulting in peptides that range from 0.5 to 3 kDa and subsequently purified with C18 resins. The enzyme trypsin is preferred due to its high specificity and cost-effectiveness; other enzymes can also be used. The samples are then separated by reverse-phase chromatography, ionized, and introduced into the spectrometer, which performs two main processes. The MS1 surveillance scan measures the mass/charge ratio (*m*/*z*) and peptide intensity, while the MS2 fragments the peptide to identify its sequence. Fragmentation occurs through collision-induced dissociation (CID), and the resulting spectra are used to identify proteins by searching databases or through de novo sequencing. Protein identification involves matching experimental spectra with theoretical ones in databases or deducing the sequence directly from the spectra. Identification algorithms compare spectra to databases containing previously digested theoretical sequences, scoring the matches. The false discovery rate (FDR) is calculated using decoy databases with random sequences to estimate accuracy [28].

#### 2.1.3. Sequential Window Acquisition of All Theoretical Mass Spectra (SWATH)

SWATH is a label-free technique that quantifies proteins using two methods—Data-Independent Acquisition (DIA) and Data-Dependent Acquisition (DDA)—in order to minimize the variability caused by chemical changes. This technique enables the acquisition of better data from samples by scanning the complete *m*/*z* range for precursor ions in a continuous and systematic process. By doing so, after the initial data collection, peptides can be reanalyzed, thus leading to more precise quantification and ultimately ensuring a high level of accuracy in the analyzed samples. Based on targeted methodologies such as selected reaction monitoring/multiple reaction monitoring (SRM/MRM), SWATH offers deeper proteomic insights, which makes it reliable in various studies across multiple research domains. It can also determine the features of sub-proteomes with low protein concentrations and assess post-translational modifications (acetylation, succinylation, and glycosylation) [29,30,31].

#### 2.1.4. Microarray Technology

First developed for DNA analysis, microarray technology was adapted to protein studies when it became clear that the mRNA (messenger RNA) profile did not always accurately represent protein expression [32,33]. Unlike the genome (which has tens of thousands of genes), the proteome is significantly more intricate, containing millions of structurally and functionally interconnected proteins. Protein microarrays allow the simultaneous analysis of many proteins in small biological samples, using recognition methods similar to enzyme-linked immunosorbent assay (ELISA) tests [34]. Several types of microarrays have been developed: analytical (antibody arrays)—based on antibodies fixed on a slide; functional—based on purified proteins, allowing the analysis of protein–protein interaction, enzymatic activities, etc.; and reverse-phase—based on cell or tissue lysates, useful in oncology for the detection of altered molecular networks. These formats allow the analysis of signaling networks involved in tumorigenesis, metastasis, treatment resistance, and immunoevasion [35,36,37,38,39]. Protein microarrays can have high density (>1000 elements)—useful for identifying new proteins and interactions—or low density (9–100 elements) for precise quantitative measurements [40,41,42]. Innovative tools, such as NAPPA (nucleic acids programmable protein array) or the multiplexed NAPPA (M-NAPPA), enable the simultaneous expression and display of thousands of proteins [43,44].

#### 2.1.5. Protein–Protein Interactions (PPIs)

Primarily formed through non-covalent interactions between amino acid side chains, PPIs are fundamental in systems biology, as proteins are involved in various processes, from intercellular communication and metabolic functions to cellular growth and overall development [45,46]. PPIs enable scientists to predict unknown proteins based on their interaction networks, as most proteins function within protein complexes. This approach enables molecular biology research to uncover key pathophysiological mechanisms that different therapies can subsequently target [47,48,49,50]. Srinivasa Rao et al. conducted a comparative study of in vitro, in vivo, and in situ PPI analysis methods for both prokaryotes and eukaryotes, showing that including more genomes in studies, along with the use of combined computational methods, enhances the accuracy of protein identification [51].

### 2.2. Proteomic Markers: Profiling, Stability, Degradation, and Forensic Implications

Due to their crucial role in evaluating the underlying pathophysiological mechanisms of cardiovascular diseases, as well as in improving clinical and prognostic outcomes, proteomic biomarkers have been intensively studied, with numerous reliable proteins being identified. Advancements in proteomic techniques enable higher diagnostic accuracy in both clinical and forensic settings, with various proteins related to cardiac function providing valuable insights into SCDs [52].

BNP (B-type natriuretic peptide) and NT-proBNP (N-terminal pro-B-type natriuretic peptide) are key proteins that myocytes secrete in response to ventricular wall stress and are widely used in cases of heart failure. Although they demonstrate high cardiac sensitivity and specificity, age and renal function can influence their actual levels and precise dosing. MR-proANP (Mid-Regional Pro-Atrial Natriuretic Peptide) and MR-proADM (Mid-Regional Pro-Adrenomedullin) can be used in parallel under heart failure conditions, as these markers are more reliable during acute episodes, offering moderate sensitivity and high specificity [53,54].

Cardiac troponins I and T (cTnI and cTnT) are considered the gold standard for diagnosing myocardial infarction because they provide a wide diagnostic window. With a positive turn in concentration within a few hours after the ischemic event, troponins offer not only high cardiac specificity but also a favorable analytical basis for dynamic value detection as their levels remain elevated for several days, which is important in both clinical and research settings [55]. In addition to troponins, CK-MB (creatin kinase MB) and LDH (lactate dehydrogenase) have traditionally been used to indicate myocardial infarction. Still, their specificity is limited. All three of the mentioned biomarkers are affected by other chronic health conditions, making it challenging to assess specific myocardial damage using them [52,56].

Another common biomarker analyzed during cardiac events is myoglobin. Although its levels rise quickly after myocardial damage, myoglobin has low cardiac specificity, due to the fact that skeletal muscle cells also release it. Ischemia-modified albumin (IMA) is also a reliable marker for detecting early ischemic events. Its level increases under oxidative stress, but may be affected by systemic conditions and liver functions [57,58,59,60,61].

C-reactive protein (CRP) is an acute-phase marker synthesized by the liver in response to inflammation triggered by cytokines. CRP, along with its highly sensitive form (hs-CRP), is widely used to monitor the progression of atherosclerotic plaques, but also to assess cardiovascular risk, particularly for myocardial infarction or stroke. Traditional risk assessments such as the Framingham score are often outweighed by hs-CRP, making it a reliable biomarker even for individuals with apparently good health. Although CRP differences can influence CRP levels, this protein remains a valuable tool in the diagnostic panel used for evaluating cardiovascular diseases [62,63,64].

Macrophage-derived Galectin-3 (Gal-3), an emerging biomarker, reflects myocardial fibrosis, particularly relevant in heart failure. Elevated levels of Gal-3 are associated with a poorer prognosis in chronic heart failure patients [65,66]. Likewise, copeptin, a peptide released alongside vasopressin, is increasingly used as a biomarker to quickly exclude myocardial infarction when combined with troponins [67,68], even though it has a small diagnostic value when used alone [69].

Among the early detection proteins, glycogen phosphorylase isoenzyme BB (GPBB) and heart-type fatty acid-binding protein (H-FABP) show rapid elevations following ischemic injury. GPBB, originating from cardiac and brain tissues, is moderately specific for myocardial ischemia. H-FABP functions as a sensitive biomarker in the early diagnosis of myocardial infarction, being released within 1 h of cardiac injury, and it has been utilized in clinical and forensic contexts to identify postmortem myocardial injury [70,71,72,73,74].

Additional plasma proteins associated with myocardial infarction have been identified through high-resolution mass spectrometry analyses. Cardiac myosin-binding protein C (cMyBP-C), plasminogen, complement C8 beta, coagulation factor II, and alpha-1-acid glycoprotein have all demonstrated significant elevations in cases of acute myocardial infarction, thereby indicating the involvement of myocardial injury, inflammation, and coagulation pathways, with cMyBP-C positioning itself as a robust emerging biomarker for its cardiac specificity and sensitivity [75,76,77,78].

Proteomic profiling identified a set of six proteins with predictive value for SCD in patients with hypertrophic cardiomyopathy (HCM): thrombospondin-1 (THBS1), complement C3 (C3), aldolase A (ALDOA), Ras suppressor protein 1 (RSU1), glutathione S-transferase omega 1 (GSTO1), and talin-1 (TLN1). This panel demonstrated high diagnostic accuracy in differentiating HCM patients at elevated risk of SCD utilizing ultra-performance LC-MS/MS [79].

From a forensic standpoint, multiple proteins provide enhanced utility in postmortem analysis. In instances of sudden, unexplained death attributed to cardiac hypertrophy, MYH7 (Myosin-7, β-Myosin heavy chain) and MYL3 (Myosin light chain 3)—both sarcomeric contractile proteins—demonstrated significant elevation relative to control subjects and other causes of cardiac death, such as neurogenic cardiac injury. These biomarkers exhibit high specificity and durability against degradation, making them suitable for postmortem differentiation of cardiac etiologies [80].

SELENBP1 (selenium-binding protein 1) and vinculin (VCL) were recently analyzed in cases of death caused by coronary artery spasm, proving their reliability in such instances. SELENBP1 showed high sensitivity and specificity rates, while VCL demonstrated discriminatory capacity [81]. Pentraxin 3 (PTX3) levels were also found to be elevated in cases of acute coronary syndrome. Secreted by macrophages and vascular smooth muscle cells, its association with thrombotic lesions is significant for future research on coronary thrombosis regarding forensic cases that exceed the capacity of histological analysis [82].

Multi-omics studies and proteomic classifiers supported by machine learning validate the value of combining these proteins into predictive models for cardiovascular outcomes and, potentially, in forensic profiling [74,83,84].

Table 1 comprehensively lists the researched proteins and relevant characteristics.

## 3. Metabolomics: Insights into Biochemical Changes

### 3.1. General Considerations

Metabolites are small molecules found in biological specimens such as plasma, tissues, or urine that change their circulating levels under various physiological and pathological conditions. The metabolomics approach is a constantly evolving field that analyzes these small molecules by dividing them into subsets with common properties (such as polarity, function, or structural similitude), proceeding to a personalized and optimized scan for each set [86].

Metabolites are the end products of cellular regulatory processes, providing a true picture of the internal metabolic balance. Therefore, their analysis directly reflects the phenotype of the organism, integrating influences from genomic, transcriptomic, and proteomic changes. To explore these small molecules, metabolomics relies mainly on nuclear magnetic resonance spectroscopy (NMR)—robust and reproducible [87,88,89]—and mass spectrometry (MS)—often coupled with separation techniques such as GC-MS (gas chromatography), ideal for volatile compounds; LC-MS, with reversed-phase (RPLC) for hydrophobic/moderately polar metabolites and HILIC (hydrophilic interaction liquid chromatography) for polar compounds; and CE-MS (capillary electrophoresis with MS), effective in the analysis of highly polar metabolites [89,90,91].

Mass spectrometry has become a reference technique in clinical laboratories due to its high specificity and sensitivity, but also due to its ability to simultaneously analyze a large number of analytes in a short time [92,93,94,95]. More recently, LC-MS/MS with high-resolution analyzers such as TOF (time-of-flight), QTOF (quadrupole time-of-flight), and Orbitrap has become the preferred choice in modern clinical settings, especially for the rapid and precise analysis of hormones, proteins, drugs, and metabolites—including point-of-care applications [92,96,97,98].

With the development of new generations of MS equipment—characterized by increased resolving power, mass accuracy, high data acquisition speeds, and molecular fragmentation capacity—it has become possible to seamlessly integrate this technology into the emerging fields of “omics” sciences, especially proteomics and metabolomics. Due to these advances, mass spectrometry-assisted metabolomics has rapidly established itself as a fundamental tool for the investigation of cardiovascular diseases, providing an integrated perspective on the metabolic alterations involved in cardiac pathology [98,99,100,101].

### 3.2. Metabolomic Markers: Main Applications and Forensic Use

With continuous improvements in necessary techniques, recent studies show metabolomic biomarkers emerging as valuable predictors in cardiovascular diseases, with potential applications in forensic medicine for assessing subsequent myocardial damage.

McGranaghan et al. conducted a systematic review of papers published between 2010 and 2019 [102]. Approximately 90% of the included studies were based on MS, highlighting the efficiency of this method in identifying metabolites relevant for cardiovascular diseases. In total, 39 biomarkers were significantly correlated with CVD (cardiovascular diseases) risk: 27 indicated as increased risk factors and 12 as protective factors. The most frequently encountered were glycerophospholipids, reported in 6 separate studies [102].

Luo J. et al. identified reliable markers in early ventricular fibrillation (VF) after ST-elevated myocardial infarction (STEMI) in their recent study [103]. By using ultra-performance LC-MS with Orbitrap on a cohort of 42-STEMI patients (21 after VF, 21 non-VF), the authors succeeded in pointing out an increased rate of 9-cis-retinoic acid (9cRA) and dehydrophytosphingosine in samples from after-VF patients, suggesting the potential of these metabolites in risk stratification for fatal events before ischemia is fully constituted [103].

Zhang et al. investigated anaphylaxis and mechanical asphyxia by metabolomic analysis of postmortem plasma using GC-HRMS (gas chromatography-high-resolution mass spectrometry) [104]. Creatinine, malic acid, and uric acid were identified as significant metabolic differentiators between deaths from anaphylaxis and those from asphyxia. Creatinine showed good postmortem stability and high potential in determining the cause of death, while malic acid and uric acid were useful but influenced by metabolic status and tissue degradation [104].

Song et al. identified 23 differential metabolites in acute myocardial infarction, including acylcarnitines, amino acids, and modified glycines (e.g., threoninyl-glycine, glutarylglycine) [105]. The metabolites were correlated with the presence of fragmented QRS waves (fQRS), indicating prognostic potential. Although the clinical relevance is high, the forensic application is limited due to rapid postmortem clearance [105].

Floegel et al. analyzed the association between acylcarnitines, amino acids, phospholipids, and hexose and the risk of myocardial infarction (MI) and ischemic stroke in two large German cohorts (EPIC-Potsdam and Heidelberg) [106]. Some sphingomyelins and phosphatidylcholines showed positive correlations with total and LDL (low-density lipoprotein) cholesterol and were significantly associated with the risk of myocardial infarction, even after adjustment for other risk factors. In contrast, no clear association was observed with the risk of stroke [106].

Paynter et al. identified 33 metabolites related to coronary artery disease (CAD) in postmenopausal women, of which 8 (such as glutamine, glutamate, cytidine monophosphate, and oxidized derivatives of arachidonic acid namely 15-HETE–15-hydroxyeicosatetraenoic acid, 5-HETE–5-hydroxyeicosatetraenoic acid, 11-HETE–11-hydroxy-5,8,12,14-eicosatetraenoic acid) remained significant after adjustment for classic risk factors [107]. C34:2-hydroxy-phosphatidylcholine was the most robust biomarker and was validated in a third dataset, which also included men [107].

Ganna et al. took an untargeted approach and found four lipid metabolites–LPC 18:1 (lysophosphatidylcholine 18:1), LPC 18:2 (lysophosphatidylcholine 18:2), MG 18:2 (monoacylglyceride 18:2), and SM 28:1 (sphingomyelin 28:1)–independently associated with cardiovascular events, validating the results in two cohorts of over 2000 people [108].

Acylcarnitines have shown consistent effects in risk prediction. Rizza et al. demonstrated that medium and long forms (e.g., C2–C18:2, medium–long chain acylcarnitines) can improve traditional risk scores such as the Framingham Risk Score [109]. Shah et al. identified 5 classes of metabolites (including acylcarnitines and branched-chain amino acids) associated with mortality in patients evaluated by cardiac catheterization [101]. The metabolomic model reclassified 27.5% of patients initially classified as intermediate risk, of whom 8.5% were correctly reclassified as high risk [101].

Würtz et al. evaluated three large population-based cohorts (FINRISK, SABRE, WHHHS), and 33 metabolites (measured by NMR) were found to be predictive of CV (cardiovascular) events [110]. Among them, phenylalanine and monounsaturated fatty acids were associated with increased risk, while docosahexaenoic acid (DHA) and omega-6 fatty acids showed protective effects. These four biomarkers were included in a validated risk score in the test cohorts [110].

Delles et al. replicated the predictive value of phenylalanine in the PROSPER and FINRISK cohorts, confirming its role in predicting hospitalization for heart failure [111,112]. Sliz et al. used NMR metabolomics on 5359 samples from PROSPER, demonstrating metabolic similarities between statin treatments and genetic inhibition of PCSK9 (proprotein convertase subtilisin/kexin type 9) [113].

The most promising class of lipid biomarkers turned out to be ceramides. Four species (Cer d18:1/16:0, d18:1/18:0, d18:1/24:0, d18:1/24:1) were combined into a risk score called CERT1 (ceramide-based risk score), already used clinically in the USA and Finland, which separates patients in four risk categories (low, moderate, increased, and high risk) [114,115,116]. Hilvo et al. improved this score by adding phosphatidylcholines to achieve more effective stratification [117]. The scores were validated in the WECAC, LIPID, and KAROLA cohorts, with over 1000 subjects each [117].

In another study, Ellims et al. showed that plasma lipidomic analysis can predict noncalcified coronary plaque burden in asymptomatic patients at intermediate risk [118]. Other contemporary markers (CIMT–carotid intima-media thickness, PWV–aortic pulse wave velocity, hs-CRP) did not show the same correlation. All 6 lipid classes analyzed (including phosphatidylcholine, GM3 gangliosides, and cholesterol esters) contained fatty acids typical of de novo lipogenesis, suggesting that this process may contribute to the development of noncalcified plaques [118].

Volani et al. analyzed plasma metabolic profiles of 36 arrhythmogenic cardiomyopathy (ACM) patients and 27 matched controls using targeted metabolomics (Biocrates AbsoluteIDQ^®^ p180 assay, BIOCRATES Life Sciences AG, Innsbruck, Austria) [119]. A total of 188 metabolites were measured, with 142 in the final analysis. The study found ACM patients have a distinct metabolome, affecting pathways like lysine degradation, tryptophan metabolism, arginine and proline metabolism, and fatty acid beta-oxidation. ADMA (asymmetric dimethylarginine)—the body’s inhibitor of nitric oxide synthesis—was higher in ACM samples, with decreased levels of nitric oxide and tryptophan. Carnitine C3, alpha-aminoadipic acid (alpha-AAA), and several phosphatidylcholines and lysophospholipids also showed lower rates in ACM patients. The findings of this study highlight that ongoing metabolic remodeling, characterized by impaired energy metabolism, endothelial dysfunction, and oxidative stress, is linked to arrhythmic pathologies, placing ADMA and alpha-AAA as promising biomarkers for evaluating physiopathological pathways in ACM patients [119].

The study of Jansen et al. aims to identify metabolic biomarkers in hypertrophic cardiomyopathy (HCM), focusing on carriers of *MYBPC3* founder variants, the most common genetic cause [120]. It compares metabolic differences between severe and mild or absent phenotypes using untargeted metabolomics with high-resolution mass spectrometry. A total of 30 severe and 30 milder cases, matched for age and sex, revealed 42 relevant metabolic peaks—36 linked to disease severity (*p* < 0.05), with 3 highly significant (*p* < 0.001). Key pathways include acylcarnitine, histidine, lysine, purines, steroid hormones, and proteolysis. Acylcarnitines like 9,12-hexadecadienoylcarnitine reflect fatty acid utilization and is associated with HCM severity. Elevated aminoadipic acid is linked to cardiac remodeling and diabetes risk. Uric acid is related to arrhythmias and mortality. 3-methylhistidine indicates myofibril breakdown. Histamine metabolites may play a role in heart failure. A metabolite linked to sex hormones suggests sex-related influences on HCM severity. Limitations include small size, cross-sectional design, non-standardized sample collection, and no multiple testing corrections, as it is exploratory [120].

A summary of the abovementioned metabolic markers is presented in Table 2.

## 4. Transcriptomics—Uncovering Gene Expression in Cardiovascular Dysfunction

The transcriptome is the set of all RNA transcripts in an organism, including long non-coding RNAs (lncRNAs), microRNAs (miRNAs), messenger RNA (mRNA), and many others. Examining these transcripts in a cell population at different temporal stages to assess the dynamic changes in gene expression can be accomplished through RT-PCR (reverse transcriptase polymerase chain reaction), microarrays, or RNA-seq (RNA sequencing). These methods provide a comprehensive view of gene expression in various pathologies and offer a future panel of drug-targeted molecules for improved clinical outcomes [121,122,123]. Recent studies highlight their role in diagnosis, prognosis, and forensic analysis.

Myocardial infarction (MI) has been intensively studied from a transcriptomic point of view in order to evaluate its underlying mechanisms. Various genes and ncRNAs have been found reliable in assessing inflammation, apoptosis, myocardial remodeling, and lipid metabolism, including *NR4A2* (nuclear receptor subfamily 4 group a member 2), *IRAK3* (interleukin-1 receptor-associated kinase 3), *IL1R2* (interleukin 1 receptor type 2), *CLEC4E* (C-type lectin domain family 4 member E), *MMP9* (matrix metallopeptidase 9), and *TNF* (tumor necrosis factor), with high levels in post-infarction samples [124,125,126,127]. Genes such as *TLR2* (toll-like receptor 2) [128], *CDKN2B* (cyclin dependent kinase inhibitor 2B) [129], *F3* (coagulation factor III) [130], *TXNIP* (thioredoxin interacting protein) [131], and *PPARGC1A* (peroxisome proliferator-activated receptor gamma coactivator 1-alpha) [132] play crucial roles in immunity, cell death, or energy homeostasis.

Among ncRNA molecules, microRNAs—such as miR-1-3p, miR-208a-3p, miR-499a-5p [133,134,135], miR-486-5p, and miR-21-5p [136,137]—and long non-coding RNAs (lncRNAs), including *SNHG1* (small nucleolar RNA host gene 1), *HIF1A-AS2* (hypoxia-inducible factor 1 alpha antisense RNA 2), *TTTY15* (testis-specific transcript, Y-linked 15), and *HULC* (highly up-regulated in liver cancer) [138,139,140], play essential roles in regulating inflammatory responses, fibrosis, and cardiac apoptosis. Additionally, specific circular RNAs (circRNAs), like *circZNF292*, *circUBAC2*, and *circSLC8A1*, are linked to the diagnosis and progression of MI [141,142], while miRNAs such as miR-223 and miR-126 showed a high predictability score for antiplatelet therapy efficacy in STEMI patients [143]. Various miRNAs are currently under research, targeting the development of personalized medicine using transcriptomics for individualized therapies. Among them, *miR-21*, *miR*-*499*, *miR-133*, and *miR-208a* are currently the most promising ones [144,145,146,147,148,149].

Sacchetto et al. conducted a study that included patients diagnosed with arrhythmogenic right ventricular cardiomyopathy (ARVC) [150]. The samples collected from these patients showed a marked upregulation of miR-185-5p in their plasma, in contrast with the control group. An area under the curve (AUC) of 0.854 suggests high reliability and diagnostic accuracy, making this microRNA a potential tool for both early detection and forensic evaluation of suspected ARVC cases [150].

Silverman et al. assessed the risk of SCD in patients with CHD (coronary heart disease) by analyzing circulating levels of several miRNAs [151]. MiR-150-5p and miR-29a-3p were found elevated in the analyzed samples, with an increased risk of a fatal cardiac outcome. Authors hypothesized that miR-30a-5p might play a protective role in these individuals. The combination of the three miRNAs allowed the identification of an increased risk of SCD of up to 4.8 times, which underlines their relevance as risk stratification tools outside the classic criteria for implantation of cardiac defibrillators. Although the exact sensitivity and specificity values were not detailed, the authors estimate a range of approximately 80–85% [151].

In a study regarding patients diagnosed with Brugada syndrome, Steinberg et al. incorporated miR-145-5p and miR-585-3p into a multivariate model, achieving highly promising results, with an AUC of 0.96 [152]. Their findings highlight a prominent predictive capacity for major cardiac events in patients afflicted with this syndrome. Furthermore, as these markers are linked to ventricular arrhythmias and resuscitated cardiac arrest, they may be of use from both a clinical and forensic standpoint, suggesting not only high diagnostic accuracy but also the potential to clarify unexplained deaths [152].

A major contribution to the field of forensic pathology was made by Yan et al., who demonstrated that four microRNAs—miR-3113-5p, miR-223-3p, miR-133a-3p, and miR-499a-5p—have high diagnostic potential in SCD, including those cases where autopsy does not reveal obvious structural lesions (autopsy-negative sudden unexplained death, SUD) [153]. The study reported AUC values between 0.7839 and 0.9043, sensitivities between 64.71% and 97.06%, and specificities of 70–100%, thus highlighting the robustness of these biomarkers. Furthermore, the combination of two of these microRNAs allowed discrimination between specific causes of sudden death, overcoming the limits of conventional histopathological methods [153].

A first study conducted by Navarre et al. investigated the microRNA profile in children undergoing chronic ventricular pacing in the context of congenital complete atrioventricular block (CCAVB) [154]. The authors identified a set of 488 microRNAs differentially expressed between children with long-term pacing and those in the control group. The study highlighted the activation of pro-fibrotic pathways (involving miR-92a, miR-130, miR-29, and miR-27) and the dysregulation of sodium and calcium channels through let-7, especially in patients with pacing for over 10 years. Also, one of the patients, who later died suddenly from ventricular fibrillation, presented a significantly different miRNA profile, supporting the prognostic value of these molecules. Therefore, these miRNAs may serve as early molecular indicators of adverse cardiac remodeling in pediatric pacemaker patients, potentially also useful in forensic medicine in cases of sudden death recorded in apparently healthy young people [154].

Yang et al. conducted a case–control study in order to investigate the expression of three circulating microRNAs in patients with acute STEMI followed for two years after primary revascularization [155]. Patients who developed major adverse cardiovascular events (cardiac death, hospitalized heart failure, reinfarction) showed decreased levels of miR-26a-5p, miR-21-5p, and miR-191-5p, which, combined with natriuretic peptides, improved risk scores by refining standard prognostic ability. Kaplan–Meier analysis of event rates indicated important differences for these miRNAs among patients with values below the median and above it. These findings confirm the potential of non-cardiac (but systemically regulatory) microRNAs to act as true prognostic indicators after infarction, with increased clinical and forensic value [155].

A comprehensive mechanistic examination of the role of miR-31-5p in suppressing myocardial hypertrophy was demonstrated by Zhao et al. [156]. Conducted on animal models (rats with abdominal aortic coarctation), the study indicates that this microRNA modulates the expression of the Nfatc2ip gene, a transcription factor involved in the activation of the β-Mhc gene, which is essential in cardiac hypertrophy. Administration of an agomir of miR-31-5p led to a significant reduction in ventricular hypertrophy at both the histological and molecular levels, suggesting real therapeutic potential. The importance of this mechanism lies in the fact that Nfatc2ip has previously been associated with maladaptive post-infarction remodeling, which places miR-31-5p at the center of a possible new molecular regulatory circuit, with clinical and legal applications in cases of sudden death associated with clinically unsuspected cardiac hypertrophy [156].

Table 3 summarizes the relevant microRNAs discussed above and their forensic values.

## 5. The Role of Genetics in Understanding and Preventing Sudden Cardiac Deaths

In recent decades, an increasing number of hereditary arrhythmogenic diseases have been identified, accounting for many cases of unexplained arrhythmias in young people with specific genetic conditions [157,158]. In pediatric populations and young adults, SCD is closely associated with genetic cardiac disorders like cardiomyopathies and channelopathies [159,160]. Advances in genetics have allowed for the identification of specific genes that predispose individuals to SCD, improving diagnosis, risk assessment, and, in some cases, enabling familial treatment [161]. Genetic testing is essential for pinpointing the molecular causes in patients suspected of hereditary arrhythmias, offering direct clinical advantages: it helps establish or refine diagnoses (even in asymptomatic carriers), helps in risk assessment, and sometimes guides treatment choices. Understanding the connections between genotype and phenotype is also key for evaluating risk and selecting suitable therapies [162].

Testing for QT syndromes is recommended for the major genes (*KCNQ1*–Potassium Voltage-Gated Channel Subfamily Q Member 1, *KCNH2*–Potassium Voltage-Gated Channel Subfamily H Member 2, *SCN5A*–Sodium Voltage-Gated Channel Alpha Subunit 5) in symptomatic patients and their family members when suspecting long-QT syndrome (LQTS) [163]. For short-QT syndrome (SQTS) and catecholaminergic polymorphic ventricular tachycardia (CPVT), testing is advised for patients and relatives, providing both diagnostic and prognostic insights [164,165,166,167]. In Brugada syndrome, testing can help confirm the diagnosis but does not significantly impact risk stratification [168]. For arrhythmogenic cardiomyopathy, testing can establish the diagnosis and facilitate family screening [169,170]. Testing should always be integrated into a multidisciplinary approach, with appropriate genetic counseling [171].

Recognizing that genetic analysis holds substantial importance in a comprehensive evaluation of SCD and noting the growing body of research investigating these pathologies, we have prepared Table 4, which summarizes the genes implicated in SCDs attributable to genetic cardiac disorders.

## 6. Integration of Multi-Omics Approaches in Postmortem Forensic Diagnostics

### 6.1. Benefits of Combined Analysis (The Multi-Omics Approach)

Multi-omics techniques allow the identification of valuable markers in both clinical and forensic medicine, and when combined, they provide increased accuracy in the final diagnosis. The need for their continued development stems from the advantages of depicting underlying biological shifts behind cardiac diseases, especially when traditional autopsy methods are inconclusive [172].

Sudden cardiac death is often the first symptom of cardiovascular diseases. In cases where hereditary channelopathies or cardiomyopathies are suspected, systems biology analysis enables a better understanding of genetic pathways and an increased accuracy in forensic diagnosis by correlating extensive data from traditional and molecular autopsies to specific arrhythmogenic phenotypes and expressing rates in affected cardiac tissue [172,173].

Cellular signaling alterations and enzyme malfunctions can be assessed by coupling proteomic with metabolomic approaches, while transcriptomics shows insights into gene responses to biochemical changes and dynamic variability. High-throughput methods provide an overview of establishing the cause of death by integrating genomic, proteomic, and metabolomic analysis from a single sample; coupled with integrated analysis, it brings complex data that increases precision in forensic investigations, especially the correct estimation of the postmortem interval, even in degraded samples [173,174,175,176].

### 6.2. Using Artificial Intelligence and Bioinformatics in Pattern Recognition

Multi-omics analyses generate massive datasets, with an enormous number of variables that require methods for separating data into different relevant classes. Methods such as PCA (Principal Component Analysis) and PLS-DA (Partial Least Squares Discriminant Analysis) are widely used in this regard. Machine learning algorithms such as random forests allow the construction of predictive methods capable of classifying samples and identifying outcomes based on the omics profile. All this data requires statistical algorithms to achieve an accurate correlation between combined omics methods and to identify common biological networks [177,178,179,180]. A bioinformatics platform, such as MetaboAnalyst, provides reproducibility and transparency for the extracted data, thereby improving pathway interpretation in multi-omics data integration, while maintaining objectivity in postmortem analyses [178].

## 7. Challenges and Limitations

### 7.1. Technical Challenges

A major challenge in using omics in forensic science is the degradation of biological molecules after death. RNA is especially vulnerable to breaking down, which restricts the use of transcriptomic techniques on older or poorly preserved samples [181]. Proteins and metabolites can also experience spontaneous oxidation or non-enzymatic modifications that compromise measurement accuracy. Adequate biological samples are crucial for retrieving reliable biomarkers. In this regard, some studies have indicated that samples like nails, hair, or bones are often more effective than deteriorated soft tissues. Additionally, manipulation and processing variability, along with external alteration factors, must be minimized through standardized sample collection procedures [173,182,183,184,185,186,187].

### 7.2. Data Interpretation and Reproducibility Issues

As stated previously, omics approaches require advanced biostatistics and bioinformatics platforms to handle the large number of variables. Although machine learning algorithms improve the accuracy of extracted data, independent studies reveal significant disparities in various marker expression levels, particularly in transcriptomics, thus highlighting the pressing need for standardized protocols for data processing and analysis. Moreover, external validation from inter-laboratory analysis should be considered in future adopted protocols to prevent systematic errors. To guarantee reproducibility, ongoing efforts in multicenter validation and the formulation of standardized protocols for each omics platform are imperative [173,175,188,189].

### 7.3. Ethical and Forensic Considerations

Handling genetic data in postmortem molecular analyses raises pressing concerns about the ethical aspects of forensic procedures, especially regarding informing the relatives of the deceased when hereditary diseases are found as a cause of death and preventive screening becomes necessary. Forensic investigations must be conducted in manners that uphold confidentiality and informed consent. Nonetheless, data acquired through omics techniques must adhere to Daubert standards for court admissibility, ensuring scientific validation, reproducibility, community acceptance, and relevance as evidence [172,188,190,191].

## 8. Conclusions

Using multi-omics in postmortem forensic diagnosis improves understanding of the mechanisms behind sudden cardiac death, especially when traditional analyses are inconclusive due to the lack of structural changes or high sample degradation. The integration of proteomic, metabolomic, and transcriptomic analysis techniques enhances the accuracy and sensitivity of autopsies by revealing changes that are undetectable through traditional methods, ultimately leading to more precise diagnoses of cardiac death.

The medicine of the future is based on a holistic approach, personalized for each patient, and forensic medicine will be no exception to this trend. Combining transcriptomic, proteomic, and metabolomic analysis techniques with bioinformatics and artificial intelligence models supports drawing parallels between expression patterns, pathways, and cause-of-death models, ultimately leading to extended individual risk profiles, especially in cases of unexplained or genetically suspected mortality. Such insights could help in developing prevention strategies, especially in families with hereditary cardiovascular diseases.

Several challenges surface in the field, with the most prominent being postmortem molecular degradation, which complicates analytical procedures. Our findings suggest that myocardium, blood, and plasma samples are preferred when the body is preserved quickly because cardiac biomarkers are measured more accurately, especially in blood samples where their integrity is less affected by autolysis compared to solid tissues and organs. When advanced decomposition occurs or only skeletal remains are available, forensic scientists should focus on more stable tissues such as nails, hair, and bones due to their lower postmortem degradation, ability to preserve molecular information for longer periods, and usefulness in proteomic, metabolomic, toxicological, and genetic analyses.

The variability in sampling methods and data processing protocols, along with increasing ethical considerations in genetic interpretation, is also a pivotal issue, particularly in cases involving heredity. Despite these limitations, several stable biomarkers with considerable forensic utility have been identified through multi-omics studies, such as cardiac myosin-binding protein C (cMyBP-C), microRNAs, and acylcarnitines, confirming the benefits of merging these techniques with traditional methods already used in autopsies.

The widespread adoption of a multi-omics approach requires standardized protocols for sampling and data processing, as well as well-established work methodologies to improve analytical precision and accuracy in both research and diagnostics. Comprehensive databases and collaborative networks should also be part of the process, taking into account the ethical aspects of data sharing. Nonetheless, wide validation studies are imperative to establishing these reliable methods as a gold standard for emerging personalized medicine, both in forensics and other medical fields, for a unique vision of the patient in each individual case.

## Figures and Tables

**Table 1:** Protein markers and individual characteristics regarding cardiac conditions—based on [16,52,79,80,81,85].

Protein	Source	Half-Life/Detection Time	Specificity and Sensitivity	Function/Role	Factors Affecting Levels	Relevance to Disease	Forensic Use
cMyBP-C [16]	Cardiac muscle	Rapid release post-MI	High cardiac specificity	Myofibrillar structural protein; released with injury	None reported	Acute MI diagnosis	Strong forensic potential
Alpha-1 Acid Glycoprotein 2 [16]	Liver (plasma)	Moderate	Moderate specificity	Acute-phase protein	Inflammation, infection	Marker in MI	Minimal forensic data
Plasminogen, Coagulation Factor II, Complement C8β [16]	Liver	Variable	Moderate specificity	Coagulation/inflammation	Inflammation, liver function	MI and thrombotic risk	Not yet validated for forensic use
BNP/ NT-proBNP [52]	Cardiac myocytes	BNP: ~20 min; NT-proBNP: ~60–120 min; elevated within hours of ventricular stretch	High sensitivity	Cardiac stress indicator	Age, renal function, obesity	Heart failure	Limited; affected by postmortem interval
Troponins (cTnI, cTnT) [52]	Cardiac myocytes	Elevated 7–10 days Detected 3–6 h post-injury	High sensitivity	Myocardial injury marker	CKD, myocarditis, sepsis	Myocardial infarction	Widely used; gold standard postmortem
Myoglobin [52]	Skeletal and cardiac muscle	Detectable in 1–3 h, peaks at 6–9 h, clears in 24 h	High sensitivity, low specificity	Early marker of muscle damage	Muscle injury, renal failure	Early detection of MI	Limited forensic value (clears quickly)
GPBB [52]	Cardiac and brain tissue	Detectable in 1–4 h, peaks at 6–12 h	High sensitivity, moderate specificity	Early ischemia detection	Pregnancy, brain injury, liver dysfunction	Early MI marker	Possibly useful if rapid sampling is possible
CK-MB [52]	Predominantly cardiac muscle	Rises in 3–6 h, peaks at 18–24 h	High specificity for MI	Energy metabolism enzyme	Skeletal muscle diseases	MI diagnosis and prognosis	Moderate (short detection window)
H-FABP [52]	Cardiac muscle	Peaks at 6–8 h, clears by 24–36 h	High sensitivity, moderate specificity	Fatty acid transport in cardiomyocytes	Skeletal muscle damage, renal dysfunction	Early MI detection	Some forensic potential (short half-life)
CRP [52]	Liver (in response to inflammation)	Rises in 6–12 h, peaks at 24–48 h	High sensitivity for inflammation, low specificity	Systemic inflammation marker	Infection, autoimmune diseases	Prognosis in CAD and HF	Limited forensic use
Complement C3 [79]	Liver (also macrophages)	Not specified	Moderate sensitivity/specificity	Inflammatory mediator	Systemic inflammation	HCM severity, SCD risk	Not directly applicable
Thrombospondin-1 [79]	Platelets, endothelial cells	Not specified	Moderate sensitivity/specificity	Angiogenesis, myocardial remodeling	Inflammation	SCD risk in HCM	Possibly useful (tissue persistence needs study)
Aldolase A [79]	Cardiac muscle	Not detailed	Not specified	Glycolysis enzyme	Non-specific stress	HCM severity	Unclear forensic value
Ras Suppressor Protein 1 [79]	Ubiquitous	Unknown	Under research	Signal transduction	Unknown	SCD correlation in HCM	Not yet validated
Talin-1 [79]	Cytoskeletal, cardiac cells	Unknown	Correlates with imaging severity	Cytoskeletal organization	Unknown	Marker for HCM disease severity	Potential tissue application
GSTO1 [79]	Liver, cardiac cells	Unknown	Moderate sensitivity	Detoxification enzyme, redox regulation	Inflammatory states	Marker in HCM and oxidative stress	Not validated yet
MYH7 [80]	Cardiac sarcomeres	Stable postmortem	High specificity	Structural protein in cardiac hypertrophy	Genetic polymorphisms	SCD due to acquired cardiac hypertrophy	Strong forensic potential
MYL3 [80]	Cardiac muscle	Stable postmortem	High specificity	Myofibrillar regulation	Mutations in HCM	Distinguishing SCH vs. CCH	Forensic utility confirmed
SELENBP1 [81]	Various (decreased in CAS)	Unknown; early marker in CAS	High sensitivity and specificity	Oxidative stress sensor	Redox-sensitive	CAS	High diagnostic accuracy postmortem
PTX3 [85]	Macrophages, smooth muscle cells	~1–2 days; early in inflammation	High sensitivity	Inflammation and damage marker	Acute inflammation	Coronary thrombosis, ACS	Useful for identifying thrombotic lesions

cMyBP-C = Cardiac Myosin-Binding Protein C; MI = Myocardial Infarction; BNP = B-Type Natriuretic Peptide; NT-proBNP = N-terminal pro B-Type Natriuretic Peptide; cTnI, cTnT = Cardiac Troponins I and T; CKD = Chronic Kidney Disease; GPBB = Glycogen Phosphorylase BB; CK-MB = Creatine Kinase–Myocardial Band; H-FABP = Heart-Type Fatty Acid-Binding Protein; CRP = C-Reactive Protein; CAD = Coronary Artery Disease; HF = Heart Failure; HCM = Hypertrophic Cardiomyopathy; SCD = Sudden Cardiac Death; GSTO1 = Glutathione S-Transferase Omega 1; MYH7 = Beta-Myosin Heavy Chain; MYL3 = Myosin Light Chain 3; SCH = Acquired Cardiac Hypertrophy; CCH = Compensated Cardiac Hypertrophy; SELENBP1 = Selenium-Binding Protein 1; CAS = Coronary Artery Spasm; PTX3 = Pentraxin-3; ACS = Acute Coronary Syndrome.

**Table 2:** Key metabolic markers for assessing myocardial function.

Study	Clinical Context	Key Metabolites	Metabolite Class	Main Application	Platform	Sample Type	Forensic Use
McGranaghan et al. [102]	Systematic review (2010–2019)	39 total (27 risk ↑, 12 protective)	Glycerophospholipids	CVD Risk	Mass spectrometry	Multiple studies	Yes
Luo J. et al. [103]	Early VF after STEMI	9cRA, Dehydrophytosphingosine	Retinoids, sphingolipids	Sudden cardiac death	UPLC-Orbitrap	Plasma	Yes
Zhang et al. [104]	Asphyxia vs. Anaphylaxis	Creatinine, Malic acid, Uric acid	Amino acids, TCA cycle	Cause of death	GC-HRMS	Postmortem plasma	Yes
Song et al. [105]	Acute MI	Acylcarnitines, modified glycines	Energy metabolism	Prognostic for MI (fQRS)	MS	Plasma	Limited
Floegel et al. [106]	MI and Stroke Risk	PCs, SMs (C16:0, C24:0, etc.)	Phospholipids	MI risk prediction	NMR	Large cohorts	No for stroke
Paynter et al. [107]	CAD in postmenopausal women	Glutamine, glutamate, CMP, 15/5/11-HETE	AA derivatives	CAD risk	MS	Plasma	Yes
Ganna et al. [108]	Untargeted CV events	LPC 18:1, 18:2; MG 18:2, SM 28:1	Lipidomics	CV events prediction	Untargeted MS	Validation cohorts	Yes
Rizza et al. [109]/Shah et al. [101]	Framingham risk, catheterization	C2–C18:2 acylcarnitines, BCAAs	Fatty acids, AA	Event risk reclassification	MS	Serum	Yes
Würtz et al. [110]	FINRISK, SABRE, WHHHS	Phenylalanine, MUFAs, DHA, ω-6 FAs	AA, lipids	CV risk prediction	NMR	Cohorts	Yes
Delles/Sliz et al. [112,113]	PROSPER and FINRISK	Phenylalanine	Aromatic AA	HF hospitalization	NMR	Large studies	Yes
Hilvo et al. [114]	CERT1 Score (ceramides)	Cer d18:1/16:0 + PCs	Ceramides, PCs	Risk stratification	MS	Plasma	Yes
Ellims et al. [118]	Noncalcified plaque in CAD	18 lipid species	PC, CE, GM3	Plaque burden	Lipidomics	Asymptomatic patients	Yes
Volani et al. [119]	Arrhythmogenic cardiomyopathy	ADMA, C3 carnitine, α-AAA	AA, carnitines, phospholipids	ACM risk	Targeted metabolomics	Plasma	Yes
Jansen et al. [120]	Hypertrophic cardiomyopathy	Acylcarnitines, uric acid, α-AAA	AA, lipids	HCM severity	Untargeted MS	Plasma	Yes

VF = ventricular fibrillation; STEMI = ST-segment elevation myocardial infarction; MI = myocardial infarction; CAD = coronary artery disease; CV = cardiovascular; CVD = cardiovascular diseases; HCM = hypertrophic cardiomyopathy; ACM = arrhythmogenic cardiomyopathy; AA = amino acids; α-AAA = alpha-aminoadipic acid; ADMA = asymmetric dimethylarginine; MS = mass spectrometry; NMR = nuclear magnetic resonance spectroscopy; PCs = phosphatidylcholines; LPC = lysophosphatidylcholine; MG = monoglyceride; SM = sphingomyelin; C2-C18:2 = medium–long chain acylcarnitines; BCAAs = branched-chain amino acids; MUFAs= monounsaturated fatty acids; DHA = docosahexaenoic acid; ω-6 FAs = omega-6 fatty acids; Cer = ceramides. ↑: Increase.

**Table 3:** MicroRNAs and forensic values in heart conditions.

Authors	miRNAs Investigated	Use Case/ Condition Studied	Specificity/ Sensitivity	Forensic Value
Sacchetto et al. [150]	miR-185-5p	ARVC diagnosis	AUC = 0.854	Early/postmortem ARVC detection
Silverman et al. [151]	miR-150-5p, miR-29a-3p, miR-30a-5p	SCD in CHD	Up to 4.8× risk when combined	Risk stratification in CHD
Steinberg et al. [152]	miR-145-5p, miR-585-3p	Brugada Syndrome	AUC = 0.96	Predicts symptoms, potential forensic biomarker
Yan et al. [153]	miR-3113-5p, miR-223-3p, miR-133a-3p, miR-499a-5p	SCD/SUD	AUC = 0.78–0.90; specificity 70–100%	Differentiation in negative autopsy deaths
Navarre et al. [154]	miR-92a, miR-130, miR-27, miR-29, let-7, miR-214, miR-210, miR-205	Chronic RV pacing in children with CCAVB and early detection of PICM	>488 miRNAs differentially expressed; several >2-fold (*p* < 0.05)	High: Identifies risk of remodeling and sudden death, even in asymptomatic cases
Yang et al. [155]	miR-26a-5p, miR-21-5p, miR-191-5p	Prediction of MACE after STEMI	Strong associations (*p* < 0.001); improved C-statistics	Moderate: Prognostic utility post-STEMI, supports postmortem analysis
Zhao et al. [156]	miR-31-5p	Cardiac hypertrophy suppression via Nfatc2ip pathway	Functional preclinical model; no ROC data	Potential: Explains sudden death from undetected hypertrophy

ST = segment elevation myocardial infarction; ARVC = arrhythmogenic right ventricular cardiomyopathy; SCD = sudden cardiac death; SUD = sudden unexplained death; CHD = coronary heart disease; CCAVB = children with congenital complete atrioventricular block; PICM = pacing-induced cardiomyopathy; AUC = area under the curve; ROC = receiver operating characteristic.

**Table 4:** Main hereditary arrhythmogenic syndromes/diseases, the genes involved, and the type of functional defect associated with each one—based on [161,162].

Syndrome/Disease	Main Genes Involved	Functional Defect
Long-QT syndrome (LQTS) [161,162]	KCNQ1, KCNH2, SCN5A, KCNE1, KCNE2, ANK2, etc.	Loss/gain of function
Short-QT syndrome (SQTS) [161,162]	KCNH2, KCNQ1, KCNJ2, CACNA1C, CACNB2	Gain/loss of functions
Brugada syndrome (BrS) [161,162]	SCN5A, CACNA1C, CACNB2, SCN10A, TRPM4, GPD1-L, SCN1B	Loss of function
Catecholaminergic polymorphic ventricular tachycardia (CPVT) [161,162]	RYR2, CASQ2, CALM1, TRDN	Abnormal calcium release from the sarcoplasmic reticulum
Arrhythmogenic right ventricular cardiomyopathy (ARVC) [161,162]	PKP2, DSP, JUP, DSG2, DSC2, DES, TMEM43	Desmosomal defects, structural and electrical damage
Hypertrophic Cardiomyopathy (HCM) [161]	MYBPC3, MYH7, ACTN2, MYOZ2, JPH2	Sarcomeric mutations, structural damage
Dilated Cardiomyopathy (DCM) [161]	TTN, LMNA, MYH7, TNNT2, DES, SCN5A	Genetically heterogeneous, structural and functional damage

KCNQ1 = Potassium Voltage-Gated Channel Subfamily Q Member 1; KCNH2 = Potassium Voltage-Gated Channel Subfamily H Member 2 (also known as hERG); SCN5A = Sodium Voltage-Gated Channel Alpha Subunit 5; KCNE1 = Potassium Voltage-Gated Channel Subfamily E Member 1; KCNE2 = Potassium Voltage-Gated Channel Subfamily E Member 2; ANK2 = Ankyrin 2; KCNJ2 = Potassium Inwardly Rectifying Channel Subfamily J Member 2; CACNA1C = Calcium Voltage-Gated Channel Subunit Alpha1 C; CACNB2 = Calcium Voltage-Gated Channel Auxiliary Subunit Beta 2; SCN5A = Sodium Voltage-Gated Channel Alpha Subunit 5; SCN10A = Sodium Voltage-Gated Channel Alpha Subunit 10; TRPM4 = Transient Receptor Potential Cation Channel Subfamily M Member 4; GPD1L = Glycerol-3-Phosphate Dehydrogenase 1–Like; SCN1B = Sodium Voltage-Gated Channel Beta Subunit 1; RYR2 = Ryanodine Receptor 2; CASQ2 = Calsequestrin 2; CALM1 = Calmodulin 1; TRDN = Triadin; PKP2 = Plakophilin 2; DSP = Desmoplakin; JUP = Junction Plakoglobin; DSG2 = Desmoglein 2; DSC2 = Desmocollin 2; DES = Desmin; TMEM43 = Transmembrane Protein 43; MYBPC3 = Myosin-Binding Protein C, Cardiac Type; MYH7 = Myosin Heavy Chain 7; ACTN2 = Actinin Alpha 2; MYOZ2 = Myozenin 2; JPH2 = Junctophilin 2; TTN = Titin; LMNA = Lamin A/C; TNNT2 = Troponin T Type 2 (Cardiac).

## Data Availability

Not applicable.
